# The Correlation of PD-L1 Expression with Tumor Budding: Evaluating the Significance of Scoring Differences in Non-Small Cell Lung Cancer

**DOI:** 10.3390/biomedicines14102338

**Published:** 2026-10-14

**Authors:** Funda Canaz, Furkan Albayrak, Güntülü Ak, Bülent Yıldız, Metin Demir, Ertuğrul Çolak, Emine Dündar, Muzaffer Metintaş

**Affiliations:** 1 Department of Pathology, Faculty of Medicine, Osmangazi University, Odunpazarı, 26040 Eskisehir, Turkey; 2 Lung and Pleural Cancers Research and Clinical Center, Department of Pulmonary Diseases, Faculty of Medicine, Osmangazi University, Odunpazarı, 26040 Eskisehir, Turkey; 3 Department of Medical Oncology, Faculty of Medicine, Osmangazi University, Odunpazarı, 26040 Eskisehir, Turkey; 4 Department of Biostatistics, Faculty of Medicine, Osmangazi University, Odunpazarı, 26040 Eskisehir, Turkey

**Keywords:** PD-L1, non-small cell lung cancer, combined positive score, tumor budding

## Abstract

**Background:** Tumor budding is a prognostic marker linked to epithelial–mesenchymal transition and the tumor microenvironment throughout various tumors. The programmed cell death protein 1 (PD-1) pathway functions as an important immune checkpoint that regulates lymphocyte activity within the tumor microenvironment. PD-1 ligand expression patterns may substantially affect the potential success of therapeutic blockade of this pathway. **Methods:** This study compares programmed cell death ligand 1 (PD-L1) expression with clinicopathological features using different scoring systems and assesses its prognostic value in patients with non-small cell lung cancer (NSCLC). **Results:** The study included 359 NSCLC cases. We found a statistically significant association between a negative combined positive score (CPS) or immune cell score (ICS) and advanced clinical stage and poor tumor differentiation. Moderate and high tumor budding scores correlated with positive PD-L1 expression across all three scoring systems. Overall survival (OS) revealed that PD-L1 positivity with ICS and CPS is a significant independent factor associated with a favorable prognosis. Kaplan–Meier analysis showed that CPS+ or ICS+ cases had significantly longer OS compared to CPS− or ICS− patients. In patients treated with immune checkpoint inhibitors, we observed no significant difference in OS between PD-L1-positive and PD-L1-negative cases across all scoring systems evaluated. **Conclusion:** Our study demonstrated a significant association between tumor budding in NSCLC and PD-L1 expression in tumor and immune cells. We showed that CPS or ICS positivity is a favorable prognostic factor for overall survival in patients with NSCLC.

## 1. Introduction

One potential therapeutic approach to activate immunity against tumors is blocking immune checkpoint pathways. Tumors can select specific immune checkpoint pathways to develop immune resistance, primarily against tumor antigen-specific T cells [1]. Extensive studies have been conducted on immune checkpoint proteins, particularly programmed cell death protein 1 (PD-1) and its ligand, programmed cell death ligand 1 (PD-L1). PD-1 is a target for immunotherapeutic agents, including nivolumab and pembrolizumab, while many other agents specifically target PD-L1. Nivolumab is a fully human IgG4 monoclonal antibody that potentiates the immune response against neoplastic cells. Treatment with nivolumab has been linked to a significant increase in antitumor activity, leading to improved response rates and prolonged survival outcomes in metastatic cancer patients who have not responded adequately to standard therapeutic interventions [2]. PD-L1, also known as B7-H1 or CD274, is expressed on myeloid, lymphoid, and normal epithelial cells, as well as on cancer cells [3]. PD-L1 has been detected in tumor cells and associated immune cells in all stages of non-small cell lung cancer (NSCLC) [4]. Immunohistochemistry demonstrates PD-L1 expression at appropriate levels and in suitable cell populations, identifying patients with metastatic NSCLC who are more likely to benefit from anti-PD-1 or anti-PD-L1 therapy. Currently, in routine clinical practice for patients with NSCLC, PD-L1 expression on tumor cells is reported as the tumor proportion score (TPS). Tumors with a TPS of 50% or higher are more likely to respond to immune checkpoint inhibitor therapy as monotherapy, whereas those with lower TPS percentages may benefit from a combination of immune checkpoint inhibitors and chemotherapy [5]. In contrast to NSCLC, other cancer types (such as gastric cancer and head and neck squamous cell carcinoma) utilize a combined positive score (CPS) that indicates PD-L1 expression in both tumor cells and immune cells in the tumor microenvironment. This scoring evaluates PD-L1 expression on tumor cells and immune cells within the tumor microenvironment. It remains unclear whether CPS may be a better predictive biomarker than TPS in NSCLC, as the current literature on this topic is quite limited [6]. Furthermore, the emerging role of PD-L1 expression on immune cells in predicting responses to immune checkpoint inhibitors has garnered considerable interest in cancer immunotherapy [7].

Tumor budding is a novel prognostic biomarker observed in solid tumors, primarily colorectal cancer [8]. Some studies highlight the relationship between tumor budding in solid cancers and epithelial–mesenchymal transition and tumor microenvironment characteristics [9,10,11]. A few studies have shown a relationship between tumor budding and clinicopathological features and prognosis in lung cancer [12]. In our literature review, we found no studies investigating the relationship between PD-L1 and tumor budding in lung cancer.

This study aims to evaluate the relationship between PD-L1, TPS, CPS, and the immune cell score (ICS) and clinicopathological findings and prognosis in patients with NSCLC.

## 2. Materials and Methods

### 2.1. Study Population and Clinicopathological Data

The study included 359 NSCLC cases diagnosed at the Pathology Department of Osmangazi University Faculty of Medicine. We recorded the following parameters: age, sex, biopsy type, smoking status, clinical stage, and treatment protocol. Microscopically, we evaluated the histological type, histological grade, number of tumor buds, and tumor budding score. We assessed tumor budding according to established guidelines and protocols for reporting colorectal cancer. After examining all tumor slides, we counted the total number of buds in a selected “hotspot” area using a 20× objective lens (HPF-equivalent area of 0.785 mm^2^). When tumor budding could not be assessed with hematoxylin and eosin (H&E) staining, specimens were examined with appropriate immunohistochemical stains (p63, clone DAK-p63, Glostrup, Denmark and TTF-1, clone SPT24, Sevilla, Spain) for diagnostic purposes. We normalized the number of tumor buds by dividing by a predetermined normalization factor. We categorized the total bud count into three groups: low (0–4 buds), medium (5–9 buds), and high (10 or more buds). We did not evaluate areas that were poorly differentiated or undifferentiated [8,11,13]. Overall survival (OS) was defined as the time from diagnosis to death. Data regarding smoking history and clinical staging for 80 patients who underwent PD-L1 Clone 22C3 pharmDx kit (Dako North America, Carpinteria, CA, USA) immunohistochemical staining, along with treatment information for 83 patients treated at various other cities or hospitals, were not available. Figure 1 provides a schematic overview of the working group.

### 2.2. Analysis of PD-L1 Expression

We identified blocks containing at least 100 tumor cells (TC) in hematoxylin-eosin sections prepared from formalin-fixed, paraffin-embedded blocks. Using an automated staining machine (Dako Omnis, Mannedorf, Switzerland), we applied the PD-L1 Clone 22C3 pharmDx kit (Dako North America, Carpinteria, CA, USA) to 4-micrometer-thick tissue sections. Tonsil tissue was used as the positive control. For TPS, any intensity of complete circumferential or partial linear plasma membrane staining of tumor cells was considered PD-L1 positive. We excluded cytoplasmic staining in tumor cells from scoring. TPS was evaluated as <1%, 1–49%, and ≥50% staining [14]. For CPS interpretation, we accepted partial or complete membrane staining of tumor cells, as well as any staining in immune cells (IC). CPS was calculated using the formula CPS = (PD-L1-positive TC + PD-L1-positive mononuclear inflammatory cells)/(total TC) × 100. This formula could yield CPS> 100, but the highest score recorded was 100. CPS of 1% and above was considered positive [7]. CPS was rated as <1, 1–19, and ≥20 [15]. Additionally, we calculated an ICS by dividing the number of ICs expressing PD-L1 by the total number of tumor cells. We defined ≥1 as positive [7]. The two pathologists jointly reviewed the stained slides using a dual-headed microscope and reached a consensus score for each case.

### 2.3. Statistical Analysis

We analyzed categorical variables using frequencies and percentages, and continuous variables using medians and ranges. We used the Mann–Whitney U test to compare groups that did not conform to a normal distribution. We used a 2 × 2 cross-tabulation (with continuity correction, Fisher’s exact test, and Pearson’s χ^2^ test) to evaluate the statistical relationship between clinicopathological parameters. We generated survival curves using the Kaplan–Meier method and assessed differences with the log-rank test. Univariate and multivariate Cox regression analyses were used to assess the effect of independent variables on survival. We conducted all biostatistical analyses using SPSS (version 23.0).

## 3. Results

### 3.1. Clinicopathological Features and PD-L1 Expression

Of the 359 patients, 170 (47.4%) had adenocarcinoma (AC), 170 (47.4%) had squamous cell carcinoma (SCC), and 2 (0.6%) had adenosquamous carcinoma (ASC). Additionally, 17 patients (4.7%) were categorized as NSCLC, not otherwise specified (NOS). The median age was 66.6 years (range, 40–91 years), with a predominance of male patients (88.3%). Tumor budding score was predominantly low (59.1%), followed by medium (26.5%) and high (11.4%), respectively. In 11 cases, tumor budding could not be assessed because the tumors were composed entirely of poorly differentiated areas. In most cases, PD-L1 was positive in both tumor and immune cells (34.8%, 125/359). Notably, PD-L1 was positive exclusively in tumor cells (TC) in 46 of 359 cases (12.8%), whereas it was found solely in immune cells (IC) in 106 of 359 cases (29.5%) (Figure 2a–f). Furthermore, staining was not observed in either tumor or immune cells in 82/359 (22.8%) cases. Table 1 and Table 2 present the clinical and pathological characteristics. Table 3 presents PD-L1 immunohistochemical staining results and scores.

### 3.2. Comparison of PD-L1 Expression Scoring with Clinicopathological Characteristics

When comparing TPS rates by gender, we found significant statistical differences, especially among those with TPS ≥ 50%, where it was 95.6% for males and 4.4% for females (*p* < 0.05). No statistical significance was found between gender and TPS+/−, CPS+/−, ICS+/−, or CPS rates. Although TPS+, CPS+, and ICS+ were more common in active smokers, no statistically significant differences were found (*p* > 0.05). The study investigated the relationship between CPS and ICS in both positive and negative conditions across different clinical stages. CPS and ICS increased significantly with disease progression (*p* < 0.05). Clinical stage did not differ significantly between TPS+/− cases or TPS rates. The investigation of the relationship between CPS+/− and ICS+/− by tumor differentiation showed that CPS+ and ICS+ were associated with moderately differentiated tumors (52%, 56.3%), whereas CPS− and ICS− were significantly higher in poorly differentiated tumors (53.7%, 57.8%) (*p* < 0.05). No relationship was found between TPS+/− and TPS rates and tumor differentiation (*p* > 0.05). We compared TBS using TPS, CPS, and ICS in both positive and negative cases. Individuals with low TBS predominantly exhibited negative scores across all scores (68.6%, 76.3%, and 71.7%). In comparison, individuals with moderate and high TBS predominantly exhibited positive scores (34.4%, 31%, and 31.6% for moderate TBS, and 13.5%, 12.7%, and 13.2% for high TBS) (*p* < 0.05) (Figure 3a–f). TBS was frequently low in cases with TPS < 1 and CPS < 1 (68.6%, 76.3%), while it was more often moderate in cases with TPS ≥ 50% and CPS ≥ 20 (39.1%, 31.2%), respectively (*p* < 0.05). No significant relationship was found between age, gender, and tumor type and TPS+/−, CPS+/−, ICS+/−, and rates of CPS. Results are presented in Table 4, Table 5 and Table 6.

### 3.3. Overall Survival

We included 276 patients in the prognosis analysis. The median OS was 19 months (95% CI: 14–23) in the TPS− group and 15 months (95% CI: 10–19) in the TPS+ group (*p* = 0.511). The median OS was the longest (19 months, 95% CI: 14–23) in the group with TPS <1, followed by the groups with 1–49 (12 months, 95% CI: 6–17) and ≥ 50 (17 months, 95% CI: 10–23) (*p* = 0.584). In CPS− and ICS− cases, the median OS was 11 months (95% CI: 5–16) and 10 months (95% CI: 6–13), respectively, while in positive cases, a significantly longer OS was observed (19 months, 95% CI: 12–15) and 26 months (95% CI: 17–34) (*p* = 0.015, *p* < 0.001). The median OS was 11 months (95% CI: 5–16) in the group with CPS < 1, 20 months (95% CI: 11–28) in those with CPS 1–19, and 19 months (95% CI: 9–28) in those with CPS ≥ 20 (*p* = 0.021) (Figure 4a–c and Figure 5a,b). In our study, the median OS for a cohort of 67 patients receiving immunotherapy was 217 days (95% CI: 93–340) for TPS− patients and 173 days (95% CI: 0.6–345) for TPS+ patients (*p* = 0.595). In this group, the median OS was shorter in CPS− (160 days, 95% CI: 1.5–318) and ICS− (160 days, 95% CI: 105–328) cases compared to CPS+ (217 days, 95% CI: 105–328) and ICS+ cases (255 days, 95% CI: 129–380), but this was not statistically significant (*p* = 0.942, *p* = 0.810, respectively). Furthermore, multivariate and univariate Cox regression analysis for OS demonstrated that PD-L1 positivity with ICS is an important independent factor for favorable prognosis (HR 0.54, 95% CI 0.39–0.74, *p* < 0.001; HR 0.54, 95% CI 0.40–0.74, *p* < 0.001). Additionally, in univariate analysis, age ≥ 65 years (HR 1.47, 95% CI 1.06–2.02, *p* = 0.019) was associated with statistically significant worse OS. In the univariate analysis, CPS was an important independent factor for a favorable prognosis (HR 0.66, 95% CI 0.46–0.93, *p* = 0.018) (Table 7).

## 4. Discussion

Lung cancer is the second most common cancer in both genders, with an estimated 226,650 cases in 2025. Despite a decline in recent years, lung cancer still surpasses other cancers, causing more deaths than colorectal, breast, and prostate cancers in 2025 [16]. Primary lung cancer arises from the lung’s epithelial cells, with NSCLC accounting for about 85% of cases. Among NSCLCs, adenocarcinoma and squamous cell carcinoma are the most common histological types [17]. Treatment for advanced NSCLC has shifted from platinum-based chemotherapy to molecular targeted therapy and immunotherapy. To this end, immune checkpoint inhibitors targeting the programmed death-ligand 1 (PD-L1)/PD-1 axis are used [18]. Chen et al. [19] observed PD-L1 expression in 57.5% of NSCLC cases (SCC 68%, AC 52%, large cell carcinoma 45%). In approximately 20–30% of NSCLC cases, PD-L1 expression has been observed to exceed 50% in both tumor cells and infiltrating immune cells [3]. Scheel et al. [4] examined PD-L1 expression in 436 genetically annotated early-stage NSCLC samples using the PD-L1 antibody 5H1. Their findings indicated that 34.4% of AC cases (88 out of 256) and 33.9% of SCC cases (61 out of 180) tested positive for PD-L1 in TC. In our study, PD-L1 was positive in 171 of 359 NSCLC patients (47.6%). Specifically, the positivity rate was 47.4% in AC, 47.4% in SCC, 0.6% in ASC, and 4.7% in NSCLC, NOS cases. Scheel et al. [4] found PD-L1 negative in 102 (39.8%) of 250 adenocarcinoma cases and 68 (37.8%) of 180 SCC cases. In this study, PD-L1 positivity was detected only in TC in 33 cases (12.9%) of AC and 20 cases (11.1%) of SCC, and only in IC in 66 (25.8%) and 51 (28.3%) cases, and in both TC and IC in 55 (21.5%) and 41 (22.8%) cases, respectively. In our study, similar to the literature, SCC and AC cases were negative in 38/170 (22.3%) cases, while NSCLC and NOS cases were negative in 6/17 (35.2%) cases. In AC and SCC cases, PD-L1 positivity was observed only in TC, at 18/170 (10.5%) and 24/170 (14.1%), respectively. Immune cells were similar at 51/170 (30%), while TC and IC were 63/170 (37.0%) and 57/170 (33.5%), respectively. Pann et al. [20] reported combined odds ratios (OR) in 1550 NSCLC cases, showing that high PD-L1 expression was associated with poor tumor differentiation [OR = 0.53, 95% confidence interval (CI): 0.39–0.72, *p* < 0.001]. In contrast, none of the other clinicopathological characteristics [gender, smoking status, histological type, tumor invasive depth, lymph node metastasis status, and tumor node metastasis (TNM) stage] correlated with PD-L1 expression in the current analysis. In this study, the combined hazard ratio (HR) suggested that high PD-L1 expression is associated with poor OS in NSCLC. Chen et al. [19] found that poor tumor cell differentiation and advanced TNM stage were associated with higher PD-L1 expression (n = 120). In this study, PD-L1-negative NSCLC patients had longer 5-year OS than PD-L1-positive patients (*p* < 0.001). PD-L1 status has been indicated as an important independent prognostic factor for NSCLC (*p* < 0.001). Velcheti et al. [21] evaluated two NSCLC case cohorts in TMA slides, comprising 340 cases from hospitals in Greece and 204 cases from Yale University. They observed PD-L1 protein expression in the tumor in 36% of cases (Greece) and 25% of cases (Yale). PD-L1 expression was significantly associated with tumor-infiltrating lymphocytes in both cohorts. PD-L1 protein expression in TC was not associated with age, gender, race, or smoking status. Patients with PD-L1 expression (both protein and mRNA) above the cutoff threshold showed statistically significantly better outcomes in both series (log-rank *p* = 0.036 and *p* = 0.027). Multivariate analysis showed that PD-L1 expression was significantly associated with better outcomes independently of histology. Few studies compare TPS, CPS, and ICS in NSCLC patients. In a study of 187 patients with advanced NSCLC undergoing immunotherapy, PD-L1 positivity was identified in 112 patients (59.9%) using TPS and in 135 patients (72.2%) using CPS. In this study, we found no significant difference in OS between TPS− and TPS+ patients (*p* = 0.20). However, CPS+ patients had longer OS than CPS− patients (*p* = 0.006) [6]. In our study, we assessed all cases using a three-point scoring system, with the highest positivity in CPS (77.2%), followed by ICS (64.3%) and TPS (47.6%). In our study, CPS− and ICS− were statistically significantly higher in poorly differentiated tumors (53.7%, 57.8%) (*p* < 0.05). We found no relationship between TPS +/− and TPS rates and tumor differentiation (*p* > 0.05). When comparing TPS rates by gender, TPS was ≥50% in males (95.6%) and 4.4% in females (*p* < 0.05). No significant relationship was found between age, gender, and tumor type with TPS+/−, CPS+/−, CPS rate, and ICS+/−. In our study, in CPS− and ICS− cases, the median OS was 11 months (95% CI: 5–16) and 10 months (95% CI: 6–13), respectively, while in CPS+ and ICS+ cases, a significantly longer OS was observed at 19 months (95% CI: 12–15) and 26 months (95% CI: 17–34) (*p* = 0.015, *p* < 0.001). There was no significant difference in OS between TPS− and TPS+ patients (*p* = 0.511). Multivariate and univariate Cox regression analyses for OS showed that PD-L1 positivity with ICS is an important independent factor for favorable prognosis (HR 0.54, 95% CI 0.39–0.74, *p* < 0.001; HR 0.54, 95% CI 0.40–0.74, *p* < 0.001). Additionally, in univariate analysis, age ≥65 years (HR 1.47, 95% CI 1.06–2.02, *p* = 0.019) was associated with statistically significant worse OS. In univariate analysis, CPS was an important independent factor for a favorable prognosis (HR 0.66, 95% CI 0.46–0.93, *p* = 0.018). Additionally, we performed univariate and multivariate analyses in patients receiving immunotherapy. Immunotherapy was not a prognostic factor in univariate and multivariate analyses. In this group, median OS was shorter in CPS− and ICS− cases than in CPS+ and ICS+ cases. However, this difference was not statistically significant (*p* = 0.942 and *p* = 0.810, respectively). Survival analyses were performed only in patients with available survival data, which represents a limitation of our study.

Imai first described tumor budding (or “sprouting”) and its association with advanced disease stage and poor prognosis in various solid cancers in the 1950s [22]. Recently, tumor buds have been defined as single cancer cells or clusters of no more than four cells, typically found around invasive tumors. Although numerous studies support the prognostic value of tumor budding, its utility as a predictive biomarker remains under investigation. Tumor budding is strongly associated with epithelial–mesenchymal transition and various factors in the tumor microenvironment. In this context, individual tumor buds interact with the tumor stroma and different components of the immune system [23]. In the exhausted immune system subtype of pancreatic cancer, high PD-L1 expression, an immunogenic tumor microenvironment rich in T cells (including Tregs), and loss of DNA mismatch repair proteins are observed. Consequently, poor outcomes for PD-L1-upregulated tumors and frequently high-grade EMT-like tumor budding are observed in tumors [10]. The exhausted immune phenotype suggests that immunosuppressive mechanisms transform a tumor microenvironment (TME) that typically does not allow tumor budding into one that does. This situation provides a potential therapeutic opportunity for PD-1 or PD-L1 inhibitors. These findings, explained by the aggressive–defensive model of tumor budding and demonstrated in CRC, suggest that composite tumor budding and immune cell scores may have added prognostic value [23,24,25]. Safaa et al. [26] demonstrated that PD-L1 expression was correlated with low tumor budding in a series of 102 gastric adenocarcinomas (GAC) and that high tumor budding and positive PD-L1 were independent predictors of short OS. They indicated that anti-PD-L1 immunotherapy may be effective in GAC with lymph node metastasis, particularly in cases with high TIL and low tumor budding.

Qian et al. [12] demonstrated in a series of 532 NSCLC cases using multivariate Cox regression analysis that tumor budding, pleural and vascular invasion, spread via air spaces, tumor size, lymph node metastasis, and tumor node metastasis are independent prognostic risk factors. Peksa et al. [27] found that in 107 cases of pancreatic adenocarcinoma with high-grade budding, tumor cells had higher PD-L1 expression (*p* = 0.008). Kadota et al. [11] showed that, in patients with stage I lung adenocarcinoma, tumor budding is an independent prognostic factor that can be used to classify recurrence risk in greater detail among patients with stage IA, stage IB, acinar-predominant, papillary-predominant, and solid-predominant tumors. This study demonstrated that high-grade tumor budding was positively associated with protumoral immune markers—stromal FoxP3+ lymphocytic infiltration, the stromal FoxP3/CD3 lymphocyte index, and tumoral IL-7R expression. Additionally, high-grade tumor budding was found to be positively associated with CD68^+^ macrophage infiltration in tumor nests and tumor-associated stroma. Several studies have reported a positive association between the density of FOXP3^+^ tumor-infiltrating lymphocytes and cancer-cell PD-L1 overexpression, another feature of an immunosuppressive tumor microenvironment [28,29]. Çakmak et al. [13] evaluated tumor budding in NSCLC by classifying it into two categories: peripheral and intratumoral. The authors found no significant differences in disease-free survival or overall survival between the two groups. In the multivariate analysis, an intratumoral budding score of 2 was identified as the only independent prognostic factor. However, the authors noted that the limited cohort size may have influenced this finding.

In our study, predominantly negative results were observed in all scores (TPS, CPS, ICS) in cases with low TBS (68.6%, 76.3%, and 71.7%), while predominantly positive results were observed in cases with moderate and high TBS (34.4%, 31%, 31.6% for moderate TBS; 13.5%, 12.7%, and 13.2% for high TBS) (*p* < 0.05). TBS was not identified as a predictive factor in univariate and multivariate Cox analyses. In our study, the limited sample size and the inclusion of patients at different disease stages may explain why TBS was not identified as a predictive factor in the univariate or multivariate Cox regression analyses. These findings highlight the need for larger, more comprehensive studies investigating the prognostic significance of tumor budding and its relationship with the tumor microenvironment in NSCLC.

## 5. Conclusions

In our NSCLC cohort, we found a significant association between increased TBS and PD-L1 positivity. Our study is the first to identify a relationship between tumor budding and PD-L1 expression in lung cancer, highlighting the need for more in-depth studies in this area. CPS and ICS negativity are associated with advanced disease and poor tumor differentiation. Kaplan–Meier survival analyses showed significantly better OS in ICS+ and CPS+ cases. Additionally, our Cox regression analyses identified ICS+ and CPS+ status as critical prognostic factors predicting advantageous outcomes. In our immunotherapy cohort, we observed longer OS in the CPS+ and ICS+ subgroups. However, this difference was not statistically significant. The small subgroup size and the inability to assess treatment–biomarker interactions limit the interpretation of our findings and highlight the need for further studies in larger cohorts.

## Figures and Tables

**Figure 1:**
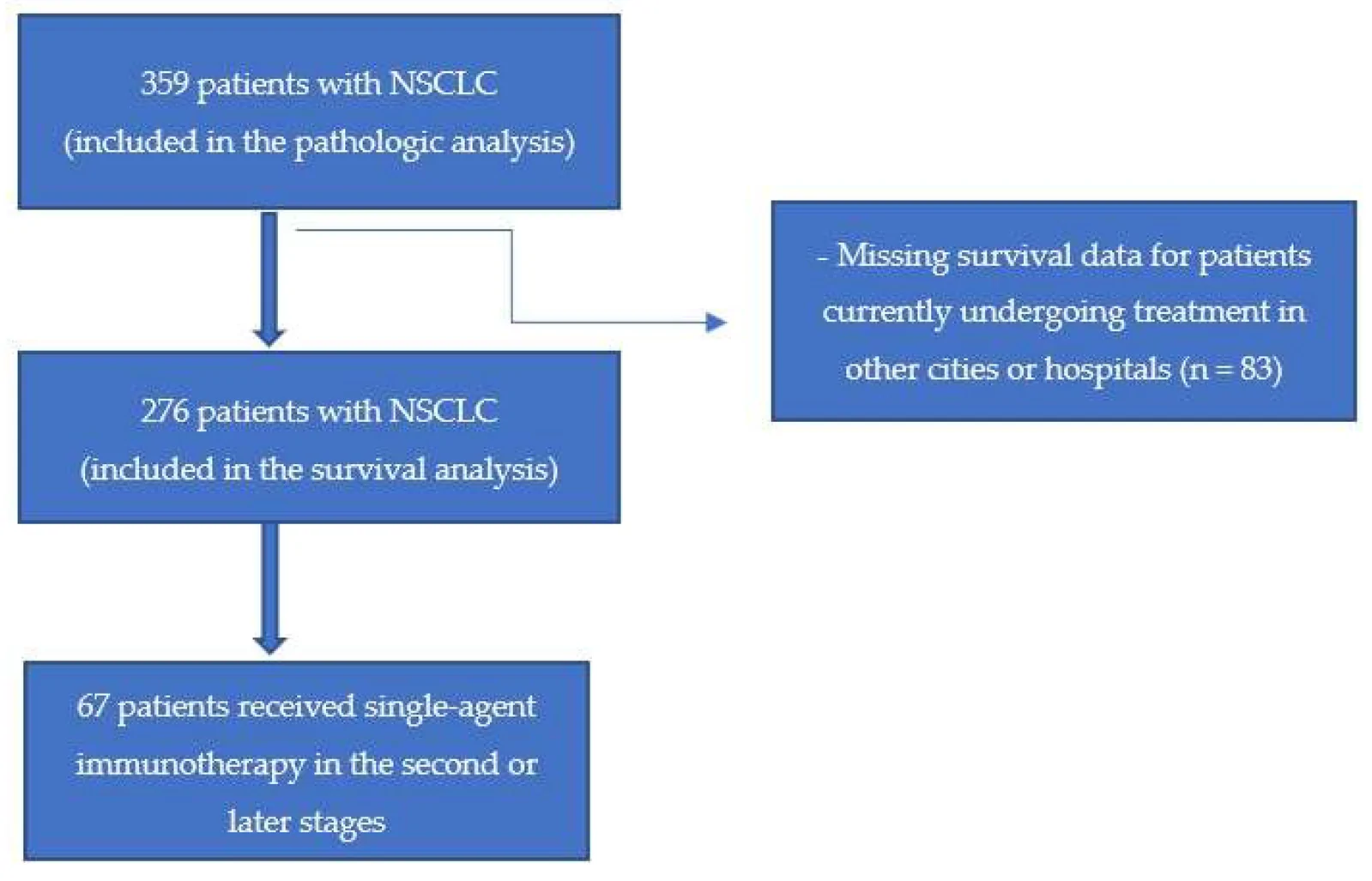
Patient enrollment flowchart.

**Figure 2:**
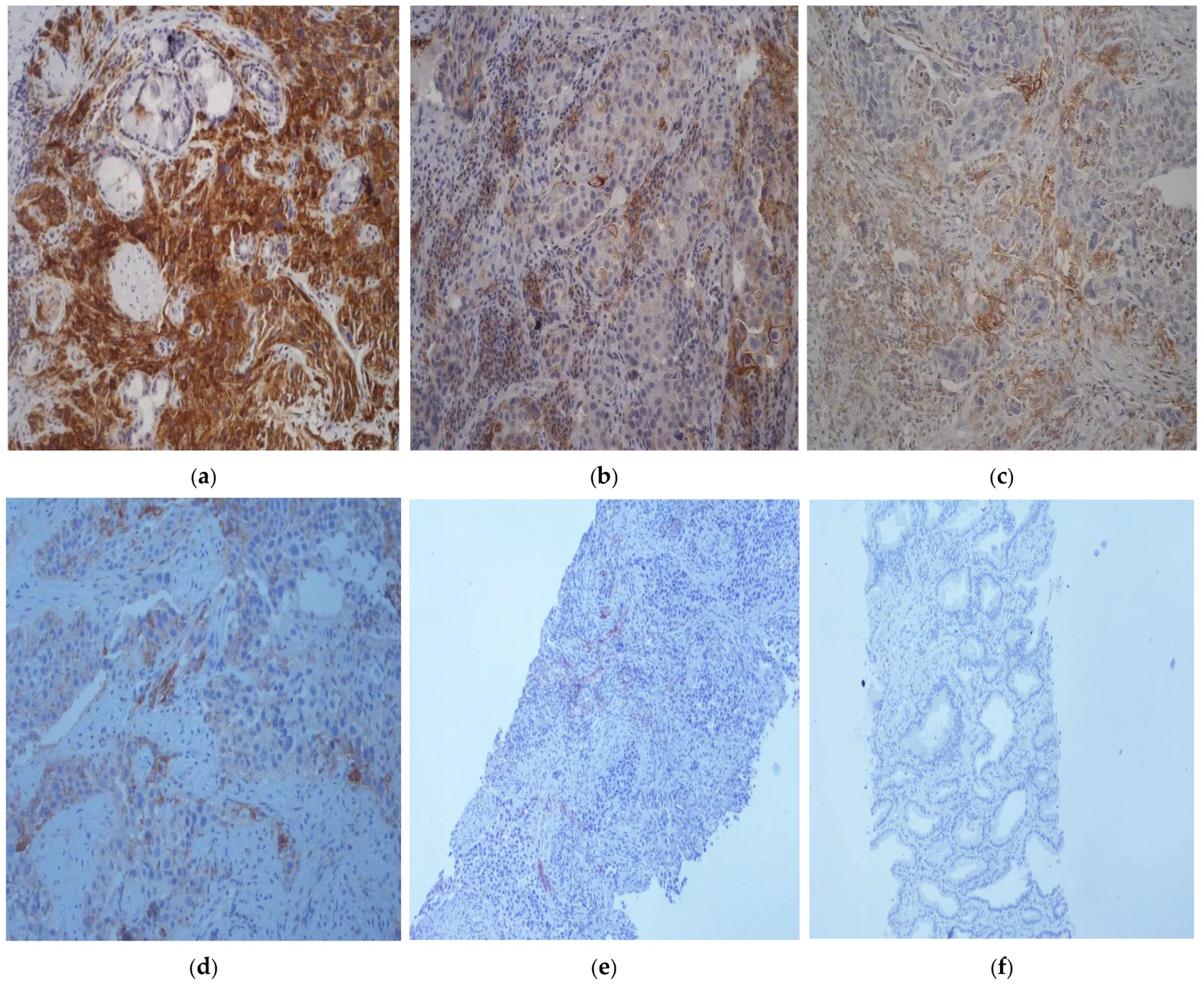
PD-L1 expression rates (membranous in tumor cells, cytoplasmic in immune cells): (**a**) in tumor cells (TPS ≥ 50%) (×200), (**b**) in tumor and immune cells (CPS ≥ 20) (×200), (**c**) in immune cells (ICS ≥ 1%) (×200), (**d**) in tumor cells (TPS 1–49%) (×200), (**e**) in tumor and immune cells (CPS 1–19) (×100), (**f**) in immune cells (ICS < 1%) (×100).

**Figure 3:**
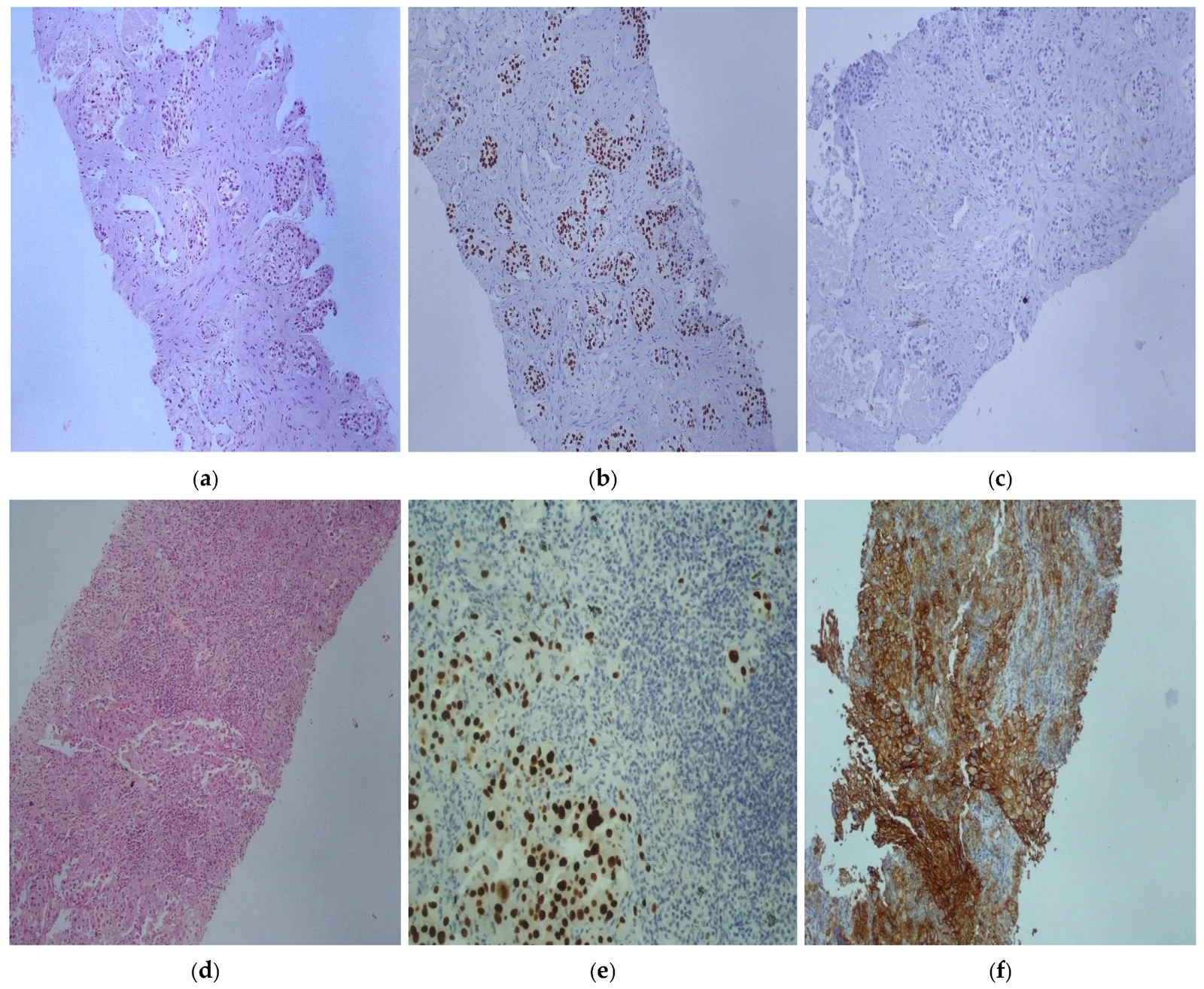
The tumor budding and immunohistochemical staining. (**a**) Low tumor budding (×100, H&E); (**b**) P63 immunochemistry (×100); (**c**) PD-L1 negative expression (×100); (**d**) high tumor budding (×100, H&E); (**e**) TTF1 immunochemistry (×200); (**f**) PD-L1 positive expression (×100).

**Figure 4:**
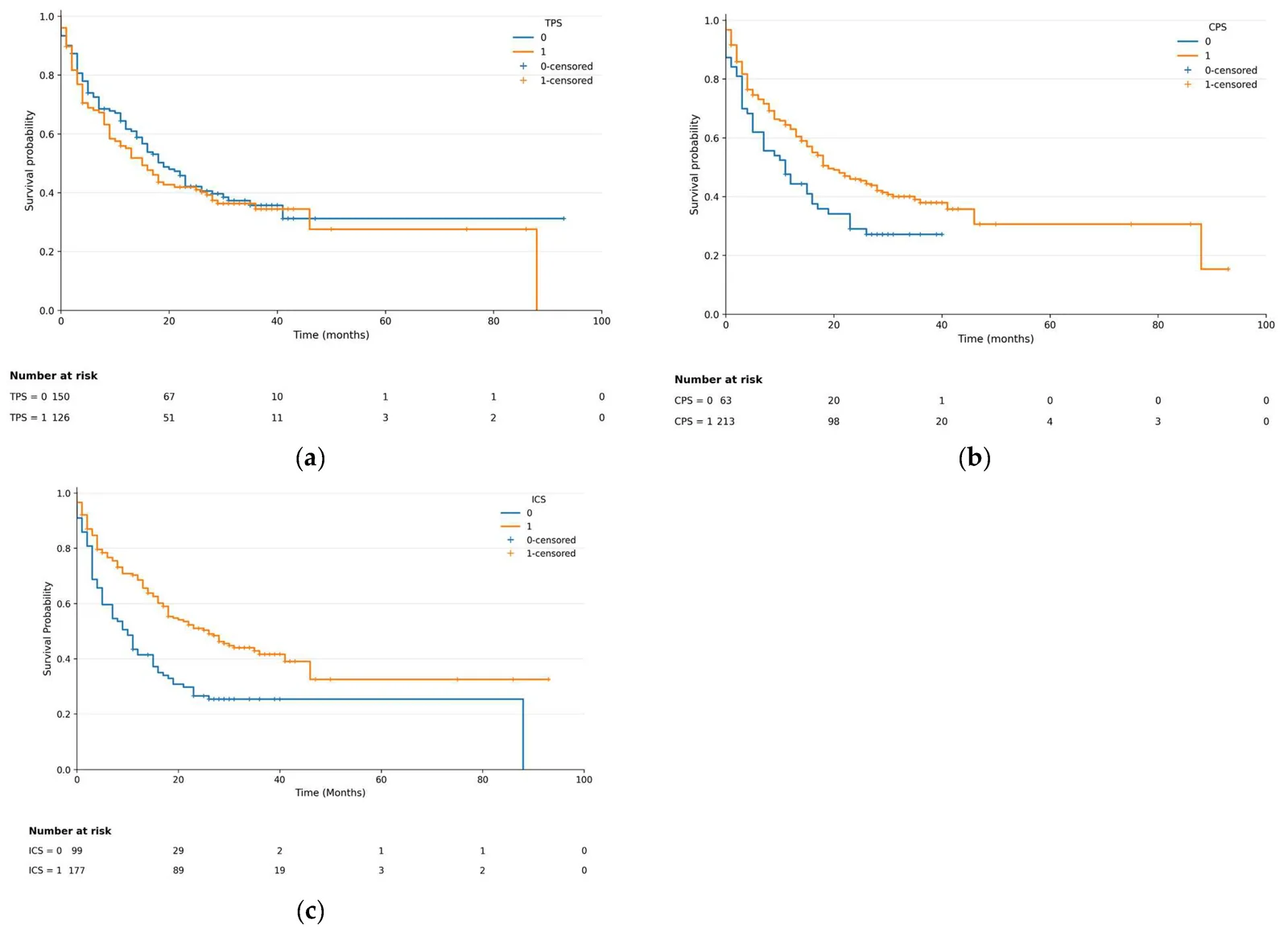
Kaplan–Meier analysis OS graph for patients with (**a**) positive TPS (orange) and negative TPS (blue), *p* > 0.05; (**b**) positive CPS (orange) and negative CPS (blue), *p* > 0.001; (**c**) positive ICS (orange) and negative ICS (blue) *p* > 0.001.

**Figure 5:**
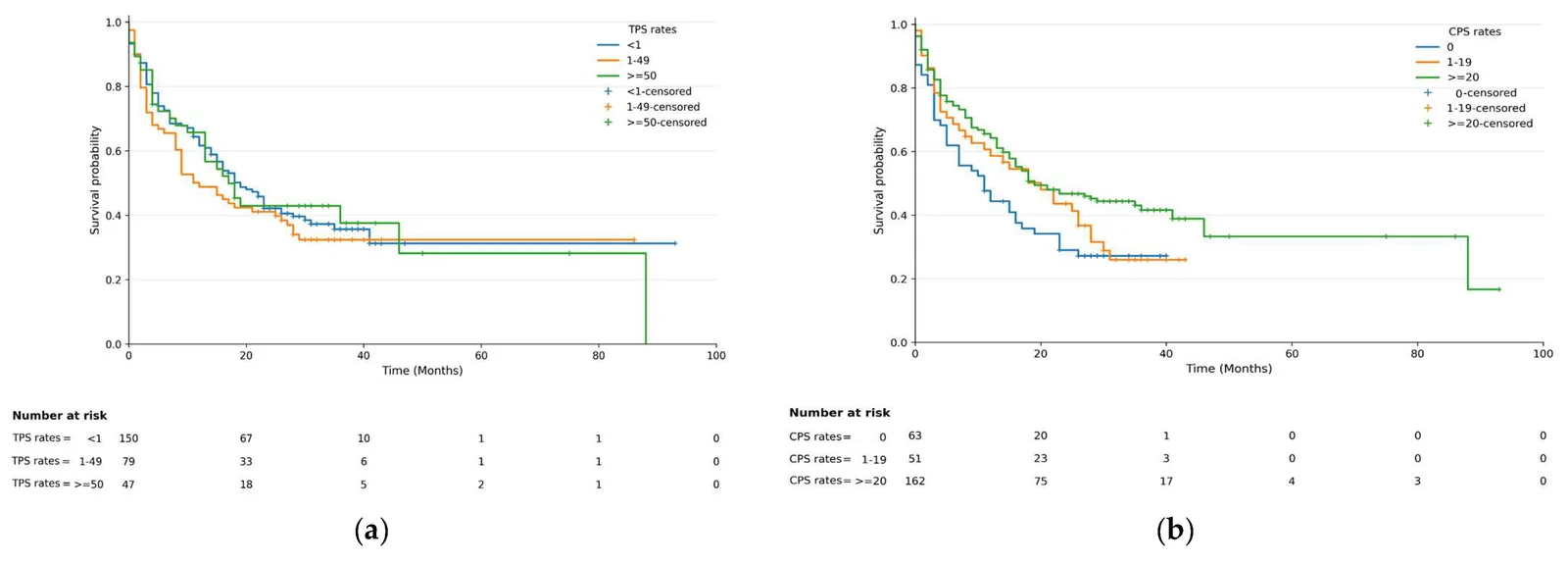
Kaplan–Meier analysis of OS graph for patients with rates. (**a**) TPS < 1% (blue), TPS 1-49% (orange), and TPS ≥ 50% (green), *p* < 0.05; (**b**) CPS < 1% (blue), CPS 1-19% (orange), and CPS ≥ 20% (green), *p* < 0.001.

**Table 1:** Demographic and clinical characteristics of patients.

Characteristics	N (%)
Age (years)	
<65	129 (35.9%)
≥65	230 (64.1%)
Gender	
Female	42 (11.7%)
Male	317 (88.3%)
Type of biopsy	
Tru-cut	300 (83.6%)
Resection	59 (16.4%)
Smoking status ^†^	
Active	167 (59.9%)
Former	79 (28.3%)
Never	27 (9.7%)
Unknown	6 (2.2%)
Clinical stage ^†^	
I–II	66 (23.7%)
III	87 (31.2%)
IV	126 (35.1%)
Line of treatments ^‡^	
First	
Surgery only	36 (13%)
Chemotherapy only	101 (28.1%)
Radiotherapy only	7 (2.5%)
Chemotherapy and radiotherapy	57 (20.7%)
Surgery and chemotherapy	17 (6.2%)
Surgery, chemotherapy, radiotherapy	3 (1.1%)
No treatment	55 (19.9%)
Second	
Immunotherapy	57 (20.6%)
Chemotherapy	28 (10.1%)
No treatment	192 (69.2%)
Third	
Immunotherapy	10 (2.8%)
Chemotherapy	16 (5.8%)
No treatment	250 (90.6%)

^†^ Information on cigarette use and clinical stage was not available for 80 patients. ^‡^ Information regarding the treatment protocol was not available for three patients. Treatment-stage information was unavailable for one patient known to be receiving immunotherapy. Consequently, this patient was excluded from the original table, which included 66 patients.

**Table 2:** Histopathologic features of cases.

Histopathologic Parameters	N (%)
Histologic type	
AC	170 (47.4%)
SCC	170 (47.4%)
ASC	2 (0.6%)
NSCLC, NOS	17 (4.7%)
Differentiation	
Well	24 (6.7%)
Moderate	174 (48.5%)
Poorly	161 (44.8%)
Tumor budding score ^†^	
Low	212 (59.1%)
Medium	95 (26.5%)
High	41 (11.4%)

Abbreviations: AC, adenocarcinoma; SCC, squamous cell carcinoma; ASC, adenosquamous carcinoma; NSCLC, NOS, non-small cell lung cancer, not otherwise specified. ^†^ In 11 cases, tumor budding could not be assessed in the biopsy specimens due to the tumors being composed entirely of poorly differentiated areas.

**Table 3:** PD-L1 expression characteristics.

PD-L1 Expression	N (%)
Cell type	
Positive only in TC	46 (12.8%)
Positive TC and IC	125 (34.8%)
Positive only in IC	106 (29.5%)
Negative in all cells	82 (22.8%)
TPS	
Positive	171 (47.6%)
Negative	188 (52.4%)
PD-L1-positive TC rates	
<1	188 (52.4%)
1–49	103 (28.7%)
≥50	68 (18.9%)
CPS	
Positive	277 (77.2%)
Negative	82 (22.8%)
PD-L1 positive TC and IC rates	
<1	82 (22.8%)
1–19	71 (19.8%)
≥20	206 (57.4%)
ICS	
Positive	231 (64.3%)
Negative	128 (35.7%)

Abbreviations: TC, tumor cells; IC, immune cells; TPS, tumor proportion score; CPS, combined positive score; ICS, immune cell score.

**Table 4:** Comparison of PD-L1 with TPS, the TPS rates, and clinicopathological findings.

	TPS		N (%)	TPS Rates			N (%)
	Positive	Negative	*p* Value	<1	1–49	≥50	*p* Value
Age (years)			0.750				0.129
<65	60 (35.1%)	69 (36.7%)		69 (36.7%)	30 (29.1%)	30 (44.1%)	
≥65	111 (64.9%)	188 (63.3%)		119 (63.3%)	73 (70.9%)	38 (55.9%)	
Gender			0.744				**0.032**
Female	21 (12.3%)	21 (11.2%)		21 (11.2%)	18 (17.5%)	3 (4.4%)	
Male	150 (87.7%)	167 (88.8%)		67 (88.8%)	85 (82.5%)	65 (95.6%)	
Smoking history			0.993				0.392
Ever	76 (60.3%)	91 (59.5%)		91 (59.5%)	48 (60.8%)	28 (59.6%)	
Former	35 (27.8%)	44 (28.8%)		44 (28.8%)	24 (30.4%)	11 (23.4%)	
Never	12 (9.5%)	15 (9.8%)		15 (9.8%)	7 (8.9%)	5 (10.6%)	
Unknown	3 (2.4%)	3 (2.0%)		3 (2%)	0 (0%)	3 (6.4%)	
Clinical stage			0.893				0.854
I–II	31 (24.6%)	35 (22.9%)		35 (22.9%)	17 (21.5%)	14 (29.8%)	
III	40 (31.7%)	47 (30.7%)		47 (30.7%)	26 (32.9%)	14 (29.8%)	
IV	55 (43.7%)	71 (46.4%)		71 (46.4%)	36 (45.6%)	19 (40.4%)	
Differentiation			0.348				0.697
Well	8 (4.7%)	16 (8.5%)		16 (8.5%)	5 (4.9%)	3 (4.4%)	
Moderately	85 (49.7%)	89 (47.3%)		89 (47.3%)	52 (50.5%)	33 (48.5%)	
Poorly	78 (45.6%)	83 (44.1%)		83 (44.1%)	46 (44.7%)	32 (47.1%)	
Histologic type			1.000				0.962
AC	81 (47.4%)	89 (47.3%)		89 (47.3%)	47 (45.6%)	34 (50.0%)	
SCC	81 (47.4%)	89 (47.3%)		89 (47.3%)	51 (49.5%)	30 (44.1%)	
ASC	1 (0.6%)	1 (0.5%)		1 (0.5%)	1 (1%)	0 (0%)	
NSCLC,NOS	8 (4.7%)	9 (4.8%)		9 (4.8%)	4 (3.9%)	4 (5.9%)	
TBS ^†^			**0.006**				**0.022**
Low	85 (52.1%)	127 (68.6%)		127 (68.6%)	54 (54.5%)	31 (48.4%)	
Medium	56 (34.4%)	39 (21.1%)		39 (21.1%)	31 (31.3%)	25 (39.1%)	
High	22 (13.5%)	19 (10.3%)		19 (10.3%)	14 (14.1%)	8 (12.5%)	

Abbreviations: AC, adenocarcinoma; SCC, squamous cell carcinoma; ASC, adenosquamous carcinoma; NSCLC, NOS, non-small cell lung cancer, not otherwise specified. ^†^ In 11 cases, tumor budding could not be assessed in the biopsy specimens due to the tumors being composed entirely of poorly differentiated areas.

**Table 5:** Comparison of PD-L1 with CPS, the CPS rates, and clinicopathological findings.

	CPS		N (%)	CPS Rates			N (%)
	Positive	Negative	*p* Value	<1	1–19	≥20	*p* Value
Age (years)			0.242				0.468
<65	104 (37.5%)	25 (30.5%)		25 (30.5%)	28 (39.4%)	76 (36.9%)	
≥65	173 (62.5%)	57 (69.5%)		57 (69.5%)	43 (60.6%)	130 (63.1%)	
Gender			1.000				0.734
Female	32 (11.6%)	10 (12.2%)		10 (12.2%)	10 (14.1%)	22 (10.7%)	
Male	245 (88.4%)	72 (87.8%)		72 (87.8%)	61 (85.9%)	184 (89.3%)	
Smoking history			0.235				0.271
Ever	133 (61.9%)	34 (53.1%)		34 (53.1%)	29 (56.9%)	104 (63.4%)	
Former	56 (26.0%)	23 (35.9%)		23 (35.9%)	18 (35.3%)	38 (23.2%)	
Never	20 (9.3%)	7 (10.9%)		7 (10.9%)	3 (5.9%)	17 (10.4%)	
Unknown	6 (2.8%)	0 (0.0%)		0 (0%)	1 (2%)	5 (3.0%)	
Clinical stage			**0.003**				**0.004**
I–II	59 (27.4%)	7 (10.9%)		7 (10.9%)	9 (17.6%)	50 (30.5%)	
III	70 (32.6%)	17 (26.6%)		17 (26.6%)	20 (39.2%)	50 (30.5%)	
IV	86 (40.0%)	40 (62.5%)		40 (62.5%)	22 (43.1%)	64 (39.0%)	
Differentiation			**0.040**				0.153
Well	16 (5.8%)	8 (9.8%)		8 (9.8%)	5 (7.0%)	11 (5.3%)	
Moderately	144 (52.0%)	30 (36.6%)		30 (36.6%)	36 (50.7%)	108 (52.4%)	
Poorly	117 (42.2%)	44 (53.7%)		44 (53.7%)	30 (42.3%)	87 (42.2%)	
Histologic type			0.545				0.635
AC	132 (47.7%)	38 (46.3%)		38 (46.3%)	31 (43.7%)	101 (49.0%)	
SCC	132 (47.7%)	38 (46.3%)		38 (46.3%)	38 (53.5%)	94 (45.6%)	
ACS	2 (0.7%)	0 (0%)		0 (0%)	0 (0%)	2 (1%)	
NSCLC,NOS	11 (4.0%)	6 (7.3%)		6 (7.3%)	2 (2.8%)	9 (4.4%)	
TBS ^†^			**0.005**				**0.016**
Low	151 (56.3%)	61 (76.3%)		61 (76.3%)	42 (60.9%)	109 (54.8%)	
Medium	83 (31.0%)	12 (15.0%)		12 (15.0%)	21 (30.4%)	62 (31.2%)	
High	34 (12.7%)	7 (8.8%)		7 (8.8%)	6 (8.7%)	28 (14.1%)	

Abbreviations: AC, adenocarcinoma; SCC, squamous cell carcinoma; ASC, adenosquamous carcinoma; NSCLC, NOS, non-small cell lung cancer, not otherwise specified. ^†^ In 11 cases, tumor budding could not be assessed in the biopsy specimens due to the tumors being composed entirely of poorly differentiated areas.

**Table 6:** Comparison of PD-L1 with ICS and clinicopathological findings.

	ICS		N (%)
	≥1 (Positive)	<1 (Negative)	*p* Value
Age (years)			0.359
<65	87 (37.7%)	42 (32.8%)	
≥65	144 (62.3%)	86 (67.2%)	
Gender			0.871
Female	28 (12.1%)	14 (10.9%)	
Male	203 (87.9%)	114 (89.1%)	
Smoking history			0.240
Ever	109 (60.9%)	58 (58.0%)	
Former	47 (26.3%)	32 (32.0%)	
Never	17 (9.5%)	10 (10.0%)	
Unkonown	6 (3.4%)	0 (0.0%)	
Clinical stage			**<0.001**
I–II	54 (30.2%)	12 (12.0%)	
III	61 (34.1%)	26 (26.0%)	
IV	64 (35.8%)	62 (62.0%)	
Differentiation			**<0.001**
Well	14 (6.1%)	10 (7.8%)	
Moderately	130 (56.3%)	44 (34.4%)	
Poorly	87 (37.7%)	74 (57.8%)	
Histologic type			0.128
Adenocarcinoma	114 (49.4%)	56 (43.8%)	
SCC	108 (46.8%)	62 (48.4%)	
Adenosquamous	2 (0.9%)	0 (0.0%)	
NSCLC,NOS	7 (3.0%)	10 (7.8%)	
TBS ^†^			**0.011**
Low	126 (55.3%)	86 (71.7%)	
Medium	72 (31.6%)	23 (19.2%)	
High	30 (13.2%)	11 (9.2%)	

Abbreviations: AC, adenocarcinoma; SCC, squamous cell carcinoma; ASC, adenosquamous carcinoma; NSCLC, NOS, non-small cell lung cancer, not otherwise specified. ^†^ In 11 cases, tumor budding could not be assessed in the biopsy specimens due to the tumors being composed entirely of poorly differentiated areas.

**Table 7:** Univariate and multivariate analyses of prognostic variables associated with overall survival.

	Univariate		Multivariate	
Variable	HR (95% CI)	*p*	HR (95% CI)	*p*
Age > 65	1.47 (1.06–2.02)	**0.019**	1.01 (0.99–1.03)	0.082
Gender	1.56 (0.94–2.71)	0.110	1.50 (0.86–2.60)	0.14
Smoking				
Ever				
Former	0.50 (0.20–1.25)	0.141	1.10 (0.77–1.59)	0.578
Never	0.61 (0.24–1.55)	0.306	0.94 (0.50–1.76)	0.863
Unknown	0.44 (0.16–1.21)	0.113	2.38 (0.85–6.67)	0.097
Immunotherapy	1.01 (0.72–1.41)	0.936	1.08 (0.75–1.54)	0.670
TPS	1.10 (0.81–1.49)	0.519	1.07 (0.78–1.48)	0.640
CPS	0.66 (0.46–0.93)	**0.018**	3.42 (0.23–50.88)	0.372
ICS	0.54 (0.40–0.74)	**<0.001**	0.54 (0.39–0.74)	**<0.001**
Tumor budding score				
Low				
Medium	1.23 (0.86–1.75)	0.240	1.26 (0.88–1.80)	0.194
High	1.37 (0.86–2.19)	0.175	1.34 (0.83–2.16)	0.220
Grade				
Well				
Moderately	0.70 (0.36–1.36)	0.299	0.69 (0.34–1.38)	0.304
Poorly	1.03 (0.53–1.98)	0.926	0.82 (0.41–1.63)	0.574

## Data Availability

The original contributions presented in this study are included in the article. Further inquiries can be directed to the corresponding author.
